# Rare Case of *Echinostoma cinetorchis* Infection, South Korea

**DOI:** 10.3201/eid3008.240289

**Published:** 2024-08

**Authors:** Sooji Hong, Hyejoo Shin, Yoon-Hee Lee, Sung-Jong Hong, So-Ri Kim, Youn-Kyoung Kim, Young-Jin Son, Jeong-Gil Song, Jong-Yil Chai, Bong-Kwang Jung

**Affiliations:** MediCheck Research Institute, Korea Association of Health Promotion, Seoul, South Korea (S. Hong, H. Shin, Y.-H. Lee, J.-Y. Chai, B.-K. Jung);; Convergence Research Center for Insect Vectors, Incheon National University, Incheon, South Korea (S.-J. Hong);; Dr. Song Jeong-Gil’s Internal Medical Clinic, Pyeongtaek, South Korea (S.-R. Kim, Y.-K. Kim, Y.-J. Son, J.-G. Song);; Seoul National University College of Medicine, Seoul (J.-Y. Chai)

**Keywords:** *Echinostoma cinetorchis*, colonoscopy, molecular identification, enteric infections, parasites, zoonoses, South Korea

## Abstract

A woman in South Korea who underwent a colonoscopy for occasional gastrointestinal discomfort had 4 adult flukes of *Echinostoma cinetorchis* showing 37 collar spines around the oral sucker recovered from the terminal ileum through the ascending colon. Partial gene sequencing showed high identity with *E. cinetorchis*.

Echinostomes are zoonotic intestinal flukes infecting birds and mammals worldwide (*1*,*2*). Adult echinostomes generally inhabit the small intestines of the definitive host and attach to the mucosal surface, causing pathological changes that include inflammation of the mucosa, bleeding, and ulceration (*1*). *Echinostoma cinetorchis* infects humans, dogs, cats, rodents, chickens, and ducks in South Korea, Japan, China, Taiwan, and Vietnam (*1*,*2*). The second intermediate hosts—that is, the source of infection for definitive hosts—include freshwater snails, fish, and amphibians (*1*). Human *E. cinetorchis* infection has been relatively rare and reported in only a few patients who had abdominal pain, diarrhea, weakness, and weight loss (*1*,*3*). We report the case of a woman in South Korea infected with *E. cinetorchis* whereby adult flukes were recovered through colonoscopy and identified by morphologic and molecular analyses.

A 69-year-old woman with occasional gastrointestinal discomfort, indigestion, constipation, and diarrhea visited Dr. Song Jeong-Gil’s Internal Medicine Clinic, Pyeongtaek, Gyeonggi, South Korea, in October 2023. Laboratory examinations revealed overall blood counts, liver function markers, renal function indicators, and lipid profiles were within reference ranges. Feces examination revealed negative results for protozoa and helminths.

Colonoscopy showed 4 actively motile adult trematodes in the mucosa of the ileum, cecum, and ascending colon (Figure 1, panel A). A physician removed the worms with grasping forceps and transferred them to the MediCheck Research Institute, Korea Association of Health Promotion (Seoul, South Korea), for morphologic and molecular identification. Two of the 4 worms were intact, and the remaining 2 were broken during the clipping of the worms. Researchers observed the intact worms by using a light microscope after fixation with 10% formalin under coverslip pressure and stained with acetocarmine (Figure 1, panels B, C).

**Figure 1:**
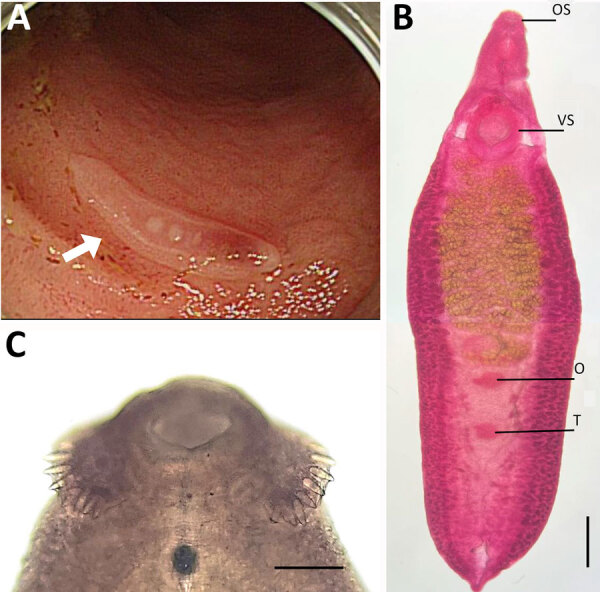
Analysis of a worm identified as *Echinostoma cinetorchis* removed during colonoscopy from a 69-year-old woman in South Korea. A) Colonoscopy image showing a moving trematode in the mucosa of the descending colon. B) Whole body of the worm. Scale bar = 0.6 mm. C) Head part of the worm showing collar spines (37 in total number) on the head collar around the oral sucker, by which it could be morphologically identified as a 37-collar-spined echinostome. Scale bar = 0.1 mm). O, ovary; OS, oral sucker; T, testis; VS, ventral sucker.

The worms were elongated and spindle-shaped, measuring ≈6.75 mm in length and 2.25 mm in width (both average measurements at the ovarian level. The worms had 37 collar spines (Figure 1, panel B), of which 24 were arranged in a single row, consisting of 6 corners and 6 laterals on each side, and the additional 13 dorsal spines were arranged in 2 alternating rows. The vitellaria were follicular and distributed mainly in lateral fields from the posterior margin of the ventral sucker to the posterior end of the body. One or both testes were absent in 3 of the 4 specimens (1 specimen had 2 testes), unlike other echinostome species, which usually have 2 testes. Intrauterine eggs (n = 10) were yellowish and operculated, measuring an average of 110 μm in length and 63 μm in width. The patient was prescribed a single dose of praziquantel (10 mg/kg).

We preserved the 2 broken worms in 70% ethanol for molecular studies. We isolated genomic DNA from the worm segments by using the DNeasy Blood and Tissue kit (QIAGEN, https://www.qiagen.com). We partially amplified (398 bp) the NADH dehydrogenase 1 (*ND*1) regions by using the standard PCR protocol with GenomicsOne 5X PCR Premix (GenomicsOne, https://www.donginbio.com) and 10 pmol of forward and reverse primers to detect *Echinostoma* spp. (*4*). We directly sequenced the PCR product at Macrogen Inc. (Seoul, Korea). Sequencing revealed 99.7% identity of our specimens (GenBank accession no. PP338757) with the sequences of *E. cinetorchis* deposited in GenBank (accession no. KU519289) (Figure 2, panel A). We obtained phylogenetic trees based on sequences of partial cytochrome c oxidase subunit 1 mitochondrial gene (*CO*1) (185bp) and internal transcribed spacer (ITS) region (ITS1–5.8S-ITS2) (657bp). Our sample for *CO*1 (accession no. PP710359) was 95.7% identical to *E. cinetorchis* (accession no. MT577587) (Figure 2, panel B), and our sample for the ITS region (accession no. PP683096) was 98.3% identical to *E. cinetorchis* (accession no. MT577832) (Figure 2, panel C).

**Figure 2:**
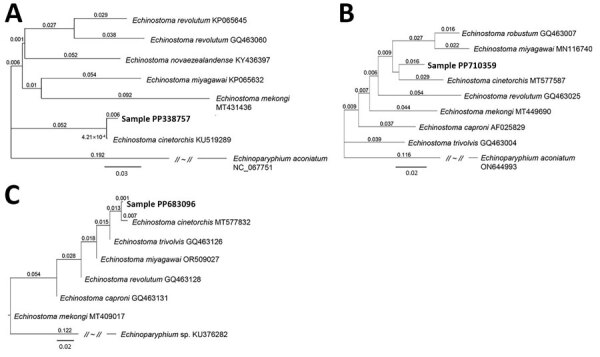
Phylogenetic trees of a worm identified as *Echinostoma cinetorchis* removed during colonoscopy from a 69-year-old woman in South Korea (bold text). Trees were based on nucleotide sequences of the NADH dehydrogenase 1 gene (A), cytochrome c oxidase subunit 1 mitochondrial gene (B), and internal transcribed spacer region (C) of the worm in comparison with various echinostome species deposited in GenBank (accession numbers shown), inferred by the neighbor-joining method (1,000 bootstrap replications) using the Geneious Prime Program 2023.1.2. (Geneious, https://www.geneious.com). *Echinoparyphium aconiatum* (NADH dehydrogenase 1 and cytochrome c oxidase subunit 1 mitochondrial genes) and *Echinoparyphium* sp. (internal transcribed spacer) were used as the outgroups. Scale bars indicate evolutionary distance.

In South Korea, few human infection cases with *E. cinetorchis* have been identified through adult worm recovery by purging with magnesium sulfate or through gastrointestinal endoscopy (*1*,*3*). Our diagnosis of *E. cinetorchis* infection was determined by adult worm recovery through colonoscopy, followed by morphologic and molecular analyses. Most adult echinostomes, such as *Isthmiophora hortensis*, reside in the upper portion of the small intestine or occasionally in the pyloric area of the stomach (*5*–*10*). In comparison, 2 endoscopy cases of *E. cinetorchis* infection (*3*), including our case, have identified the presence of worms in the colon. This unique location of echinostome flukes in humans might be a distinguishing feature for *E. cinetorchis* infection.

Freshwater snails are first as well as second intermediate hosts for *E. cinetorchis* (*1*). Large-sized snail species in particular (e.g., *Cipangopaludina*) and freshwater fish are potential sources of human infections. Our patient reported that she had sold snails and freshwater fish on the street and often consumed them raw or undercooked. Thus, the infection source for our patient might have been 1 or both kinds of intermediate hosts.

In countries where human echinostome infections are found, physicians should include echinostomiasis among the differential diagnoses of diseases causing nonspecific gastrointestinal problems. Public education regarding the hazards associated with consuming raw or undercooked snails or fish in these regions also would be useful in reducing *E. cinetorchis* infections.
